# The Association Between Pediatric COVID-19 Vaccination and Socioeconomic Position: Nested Case-Control Study From the Pedianet Veneto Cohort

**DOI:** 10.2196/44234

**Published:** 2023-02-01

**Authors:** Erich Batzella, Anna Cantarutti, Nicola Caranci, Carlo Giaquinto, Claudio Barbiellini Amidei, Cristina Canova

**Affiliations:** 1 Unit of Biostatistics, Epidemiology and Public Health Department of Cardio-Thoraco-Vascular Sciences and Public Health University of Padova Padua Italy; 2 Division of Biostatistics, Epidemiology and Public Health Department of Statistics and Quantitative Methods University of Milano-Bicocca Milan Italy; 3 National Centre for Healthcare Research and Pharmacoepidemiology University of Milano-Bicocca Milan Italy; 4 Regional Health and Social Care Agency Emilia-Romagna Region Bologna Italy; 5 Department for Women's and Children's Health University of Padua Padua Italy

**Keywords:** SEP, socioeconomic position, quantile-g-computation, nested case-control study, COVID-19 vaccine, children, area deprivation index

## Abstract

**Background:**

The success of pediatric COVID-19 vaccination strongly depends on parents' willingness to vaccinate their children. To date, the role of socioeconomic position (SEP) in pediatric COVID-19 vaccination has not been thoroughly examined.

**Objective:**

We evaluated the association between COVID-19 vaccination and SEP in a large pediatric cohort.

**Methods:**

A case-control study design nested into a pediatric cohort of children born between 2007 and 2017, living in the Veneto Region and followed up to at least January 1, 2022, was adopted. Data on children were collected from the Pedianet database and linked with the regional COVID-19 registry. Each child vaccinated with at least one dose of any COVID-19 vaccine between July 1, 2021, and March 31, 2022, was matched by sex, year of birth, and family pediatrician to up to 5 unvaccinated children. Unvaccinated children with a positive outcome on the swab test within 180 days before the index date were excluded from the analyses. Children were geo-referenced to determine their area deprivation index (ADI)—a social and material deprivation measure calculated at the census block level and consisting of 5 socioeconomic items. The index was then categorized in quintiles based on the regional ADI level. The association between ADI quintiles and vaccination status was measured using conditioned logistic regression models to estimate odds ratios and the corresponding 95% CIs. Quantile-g-computation regression models were applied to develop a weighted combination of the individual items to estimate how much each component influenced the likelihood of vaccination. All analyses were stratified by age at vaccination (5-11 and 12-14 years).

**Results:**

The study population consisted of 6475 vaccinated children, who were matched with 32,124 unvaccinated children. Increasing area deprivation was associated with a lower probability of being vaccinated, with approximately a linear dose-response relationship. Children in the highest deprivation quintile were 36% less likely to receive a COVID-19 vaccine than those with the lowest area deprivation (95% CI 0.59-0.70). The results were similar in the 2 age groups, with a slightly stronger association in 5-11–year-old children. When assessing the effects of the weighted combination of the individual items, a quintile increase was associated with a 17% decrease in the probability of being vaccinated (95% CI 0.80-0.86). The conditions that influenced the probability of vaccination the most were living on rent, being unemployed, and being born in single-parent families.

**Conclusions:**

This study has shown a significant reduction in the likelihood of receiving a COVID-19 vaccine among children living in areas characterized by a lower SEP. Findings were robust among multiple analyses and definitions of the deprivation index. These findings suggest that SEP plays an important role in vaccination coverage, emphasizing the need to promote targeted public health efforts to ensure global vaccine equity.

## Introduction

Given the high contagiousness of SARS-CoV-2 and the constant development and selection of new variants with increased pandemic potential, the success of population-based vaccination campaigns strongly depends on vaccination coverage [1]. It is largely accepted that COVID-19 vaccines are highly effective in reducing severe disease outcomes in adults, and numerous studies suggest they are also safe and effective in children [2-4]. In Italy, the first COVID-19 vaccines were approved for individuals aged between 12 and 15 years in May 2021 [5]. Since December 2021, the national drug regulation agency (Agenzia italiana del farmaco) has authorized the administration of a reduced dose of the vaccine also for children aged 5 to 11 years [5]. In December 2022, first dose coverage was 28.9% among children aged 5-11 years and 78.8% in those aged 12-19 years (information on children aged 12-14 years was not available) [6]. Before the end of the study period (March 2022), most of those first doses had been administered (with more than 95% coverage in both age groups) [6].

COVID-19 strongly impacts children from numerous perspectives, including interference with social interactions and mental health [7]. Children with COVID-19 are also at increased risk of developing multisystem inflammatory syndrome, and COVID-19 vaccines seem to reduce this risk [8,9]. Therefore, the importance of vaccination among children should not be ignored, in addition to the role played by the pediatric population in the COVID-19 transmission chain.

Depending on the characteristics of new SARS-CoV-2 variants and the mutating epidemiological scenario, future studies on vaccine effectiveness and safety in children will determine if vaccinating the pediatric population will remain a priority [1]. In 2019, the World Health Organization identified vaccine hesitancy as one of the 10 greatest threats to global health [10]. Some studies have suggested that a lower socioeconomic position (SEP) may be associated with a reduced chance of receiving a COVID-19 vaccine in adults [11-13]. However, evidence in children is scarce [14-22] also due to the shorter time since the approval of most COVID-19 vaccines. Furthermore, there are limitations to the generalizability of these works related to low numbers in the study populations and analyses exclusively based on referred data (ie, parental propensity to vaccinate their children or reported vaccine status), as opposed to actual recorded vaccination.

The surge of new variants could severely endanger public health by effectively eluding the immune system of previously immunized people, including children, increasing the population at risk of being infected and facing the consequences of COVID-19. If this were to occur, a prompt public health response would be required, and a vaccination campaign with booster doses or updated vaccines against new variants might have to rapidly reach very high portions of the population and potentially also the pediatric population. Therefore, identifying the socioeconomic determinants that most heavily affect the chances of children being vaccinated is crucial. Reducing these determinants’ effects by increasing and diversifying vaccination campaign approaches and facilitating access to vaccination centers in specific at-risk groups should become a public health priority [7,23-26].

The area deprivation index (ADI) is a composite index that has been extensively used to examine socioeconomic status [27,28]. Having low education, being unemployed, living on rent, living in crowded households, and being born in single-parent families in the area are the items that compose the index and are among the most relevant socioeconomic determinants.

The aim of this study was to examine the association between ADI and its specific components with COVID-19 vaccine status in a large cohort of children aged 5-14 years in northern Italy. Given that socioeconomic variables are strongly correlated with one another, a weighted combination of the indicators (weighted deprivation index [WDI]) was also generated to assess which components most influence the likelihood of vaccination.

## Methods

### Setting and Study Design

A case-control study design nested into a large pediatric cohort of children living in the Veneto region, Italy, was adopted. Children were defined as being vaccinated if they had received at least one vaccine dose by the end of follow-up. Vaccination status in children aged 5-14 years was examined between July 1, 2021, and March 31, 2022. Each vaccinated child was matched by sex, year of birth (from 2007 to 2017), and family pediatrician (FP; among the 33 doctors included in the analyses) to up to 5 unvaccinated children. Based on the residential status of each child, an ADI was computed at the census block level.

Data on all children included in the study derive from the Veneto Pedianet database [29]. Pedianet is an Italian network of more than 400 FPs who use an established pediatric primary care database based on the Junior Bit software in their clinical practice. Data generated by Pedianet FPs are anonymized, in compliance with Italian regulations, stored in a protected “cloud” under a unique numerical identifier, and regularly checked for validation and quality control. The database includes patient demographic and clinical characteristics, containing diagnoses (free text or coded diagnoses using the International Classification of Diseases, Ninth Revision, Clinical Modification system), drug prescriptions (recorded in accordance with the Anatomical Therapeutic Chemical codes), health care copayment exemptions, specialist visits, diagnostic procedures, hospital admissions, growth parameters (repeated measures of height and weight in accordance with national indications), and free text to report symptoms or other medical observations related to the visit. Moreover, through the electronic health record of each child included in the cohort, we were able to link the Veneto Pedianet database with the regional COVID-19 registry—where results from COVID-19 swab tests are recorded—and the regional immunization database, for vaccination history.

Inclusion in the Pedianet database is voluntary; parents or legal guardians provided consent for their children’s anonymized data to be used for research purposes in accordance with national and international regulations.

Pedianet is a large database rich in numerous clinical variables, but information on parental SEP is often missing or incomplete. SEP was first measured by means of an ADI, computed at the census block level retrieved from the 2011 Italian Census [27] (with a median number of residents of 66, IQR 28-181, per census block).

The ADI is based on 5 items that recurrently describe social and material deprivation: (1) low education, (2) unemployment, (3) living on rent, (4) crowded households, and (5) single-parent families [27,28]. The index is calculated as the sum of standardized indicators. The index was then categorized in quintiles based on the regional ADI level to ensure within-region appropriately represented categories.

The addresses of the children included in the study population were geo-referenced and linked to the census block of each Italian municipality. Through record linkage with the census block number, the ADI and all the variables defined at the census block level were retrieved for each address.

### Ethical Considerations

This is an observational, retrospective, noninterventional study. According to a bylaw on the classification and implementation of observational drug-related research, as issued by the Italian National Drug Agency (an entity belonging to the Italian Ministry of Health), this study does not require approval by an ethics committee in Italy (Italian Drug Agency note on August 3, 2007). This study was conducted in accordance with the tenets of the Declaration of Helsinki and was compliant with the European Network of Centres for Pharmacoepidemiology and Pharmacovigilance’s *Guide on Methodological Standards in Pharmacoepidemiology*.

Ethical approval of the study and access to the database was approved by the Internal Scientific Committee of So.Se.Te. Srl, the legal owner of Pedianet.

### Study Population

We identified 160,720 children from the Veneto Pedianet database. Exclusion criteria were (1) children born before 2007 or after 2017, (2) those who had the end of follow-up or the last contact with the FP before January 1, 2022, (3) those who were not properly geo-referenced or for whom it was not possible to determine an ADI, and (4) those for whom individual linkage with the COVID-19 registry was not possible (Figure S1 in Multimedia Appendix 1, details provided in Multimedia Appendix 2). Of the 25,635 children aged 5 to 14 years included in the study, 6491 (25.3%) received a COVID-19 vaccine between July 2021 and March 2022, and 19,144 (74.7%) did not. For the purpose of our analyses, we defined children as vaccinated if they received at least one dose of any COVID-19 vaccine.

**Figure 1:**
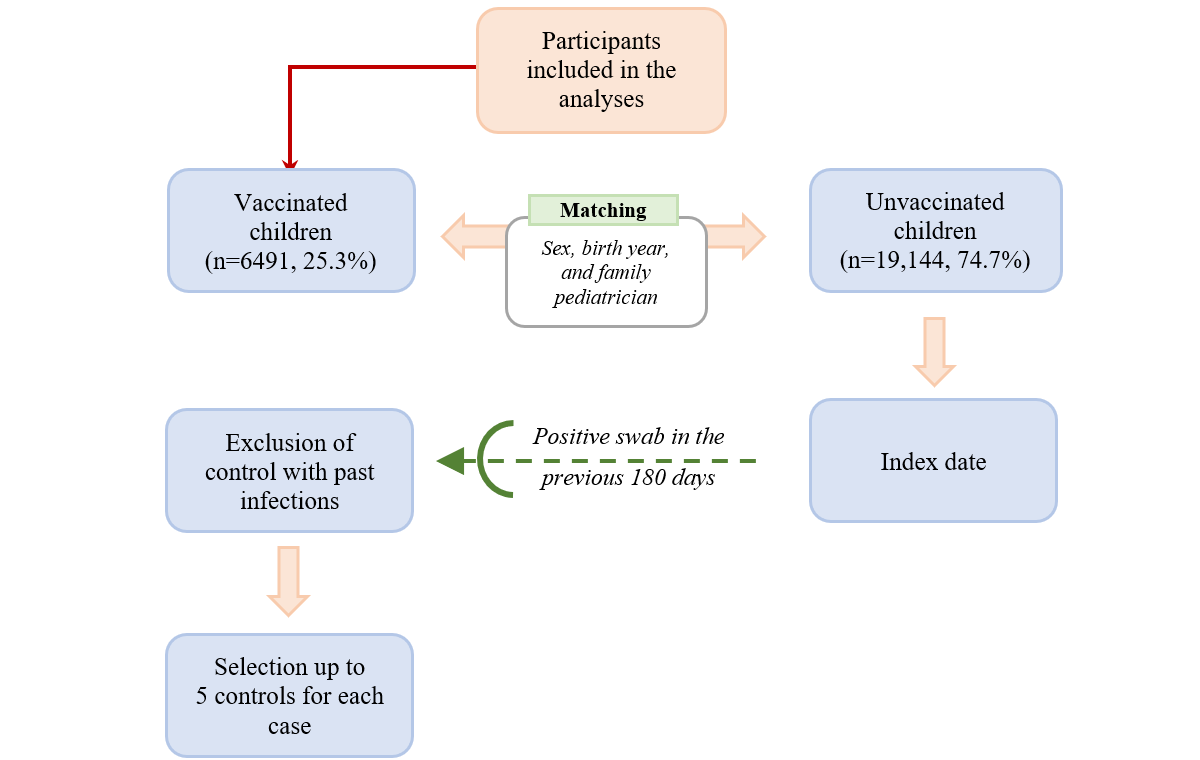
Flowchart of the study design.

Each vaccinated child was first matched by sex, year of birth, and FP to all possible available unvaccinated children. The same control was eligible for more than one case. Each control was then assigned an index date that corresponded to the date of COVID-19 vaccination of the matched case. Since matching by year of birth has led some controls to have a different age (±1 year) compared to cases, only controls who belonged to the same age group as cases at the index date (5-11 years and 12-14 years) were included. Given the indication of government agencies to vaccinate children with a previous SARS-CoV-2 infection only after 6 months, unvaccinated children with a positive swab within 180 days before the index date were excluded from the analyses. Finally, for each vaccinated child (case), up to 5 controls were randomly selected from the matched unvaccinated children (Figure 1). Matching was implemented to control for confounding and improve statistical efficiency.

### Statistical Analysis

Spearman rank correlation (ρ) was used to describe pair-wise relations between the ADI items.

Conditional logistic regression models were fitted to estimate odds ratios (ORs) and the corresponding 95% CIs for receiving a COVID-19 vaccine associated with ADI and the single items included in the index. All variables were treated as categorical with the first quintile (least deprived) as a reference. Age, sex, and FP confounding were controlled for by the study design.

To account for the strong correlation between the socioeconomic determinants that composed the ADI, we then applied a quantile-g-computation regression model to develop a weighted combination of socioeconomic variables (weighted deprivation index [WDI]) to examine its association with the outcome. Quantile-g-computation is a parametric, generalized linear model–based implementation of g-computation, which quantifies the expected change in the outcome, given a single quantile change in all exposures (the items included in the ADI) simultaneously while controlling for covariates. This method first transforms all exposures into quintiles and then fits a linear model with the dependent variables. Finally, weights are defined for each exposure, corresponding to the partial effect attributed to a specific exposure. Following these steps, the ADI items were combined to calculate a weighted index that was then categorized in quintiles. Both positive and negative relations between each indicator of the index and the outcome were allowed without any directional homogeneity assumption [30]. We defined as an item of concern any socioeconomic determinant contributing to the overall effect with a negative weight that exceeded the threshold of *1/p* (where *p* varied depending on the number of items with estimated negative weight in each stratum).

As a sensitivity analysis, the prevalence of foreigners in the area was also included among the area-based socioeconomic variables under study. All analyses were stratified by age at the index date (5-11 years and 12-14 years). Analyses were performed using SAS [31] and R [32] statistical software. We used *survival* [33] and *qgcomp* [30] packages to conduct conditional logistic regression and quantile-g-computation with R, respectively. Results with estimated *P* values of <.05 were considered significant.

## Results

### General Characteristics

The general characteristics of the study population are reported in Table S1 in Multimedia Appendix 1. Among the 25,635 children included in the study, the proportion of those who were vaccinated against COVID-19 was markedly higher for older children. The prevalence of vaccinated children varied largely based on the pediatrician and birth year (Table S1 in Multimedia Appendix 1). The distribution of the ADI quintiles of the children included in the study did not differ from the regional ADI distribution (Table S2 in Multimedia Appendix 1).

Among the specific items that composed the deprivation index, we observed strong correlations, especially between living on rent and being unemployed (ρ=0.31), followed by living on rent with crowded households (ρ=0.26). When including the presence of non-Italian ethnicity in the area, in the correlation matrix, an even stronger correlation was observed with living on rent (ρ=0.68), being unemployed (ρ=0.36), and living in crowded households (ρ=0.26).

The analyzed population consisted of 6475 vaccinated children, 3694 aged 5-11 years and 2781 aged 12-14 years, matched with 18,429 and 13,695 unvaccinated children, respectively.

### Association With Vaccination Status

Results from the conditioned logistic regression models are shown in Table 1.

This analysis revealed negative associations between the probability of being vaccinated and increasing ADI quintiles, with approximately a linear dose-response relationship. Children in the highest deprivation quintile were 36% less likely to be vaccinated against COVID-19 than those living in the least deprived areas (OR 0.64, 95% CI 0.59-0.70). The results were similar in the 2 age groups, with more pronounced differences among 5-11–year-old children. For each quintile increase of ADI, the chances of being vaccinated against COVID-19 decreased by 10% (OR 0.90, 95% CI 0.88-0.91; Table 2).

**Table 1:** Association between the quintiles of area-deprivation index (ADI) and COVID-19 vaccination status, stratified by age groups (N=38,599).

Quintile of ADI		Vaccinated (n=6475), n (%)	Unvaccinated (n=32,124), n (%)	Odds ratio (95% CI)^a^
**Total**				
	ADI 1	1501 (23.2)	6160 (19.2)	1 (reference)
	ADI 2	1332 (20.6)	5909 (18.4)	0.93 (0.86-1.02)
	ADI 3	1234 (19.1)	6291 (19.6)	0.81 (0.74-0.88)^b^
	ADI 4	1281 (19.8)	6717 (20.9)	0.77 (0.71-0.84)^b^
	ADI 5	1127 (17.4)	7047 (21.9)	0.64 (0.59-0.70)^b^
**Age group of 5-11 years (n=22,123)**				
	ADI 1	866 (23.4)	3507 (19.0)	1 (reference)
	ADI 2	764 (20.7)	3297 (17.9)	0.94 (0.85-1.05)
	ADI 3	683 (18.5)	3383 (18.4)	0.82 (0.73-0.91)^b^
	ADI 4	715 (19.4)	3971 (21.5)	0.72 (0.64-0.80)^b^
	ADI 5	666 (18.0)	4271 (23.2)	0.62 (0.55-0.69)^b^
**Age group of 12-14 years (n=16,476)**				
	ADI 1	635 (22.8)	2653 (19.4)	1 (reference)
	ADI 2	568 (20.4)	2612 (19.1)	0.92 (0.81-1.05)
	ADI 3	551 (19.8)	2908 (21.2)	0.80 (0.70-0.91)^b^
	ADI 4	566 (20.4)	2746 (20.1)	0.86 (0.76-0.98)^b^
	ADI 5	461 (16.6)	2776 (20.3)	0.67 (0.59-0.77)^b^

^a^Odds ratios and 95% CIs, estimated by means of a conditional logistic regression model.

^b^*P*<.001.

**Table 2:** Association between a quintile increase of deprivation index and COVID-19 vaccination status, stratified by age-group.

Interquintile increase in the deprivation index	Total (n=38,599), odds ratio (95% CI)	Age group of 5-11 years (n=22,123), odds ratio (95% CI)	Age group of 12-14 years (n=16,476), odds ratio (95% CI)
IQ ADI^a^	0.90 (0.88-0.92)^b^	0.88 (0.86-0.91)^b^	0.92 (0.89-0.95)^b^
IQ WDI^c^	0.83 (0.80-0.86)^b^	0.81 (0.77-0.85)^b^	0.84 (0.80-0.89)^b^
IQ WDI^d^	0.82 (0.79-0.85)^b^	0.81 (0.77-0.85)^b^	0.83 (0.79-0.88)^b^

^a^From the conditional logistic regression model; ADI: area deprivation index.

^b^*P*<.001.

^c^From the quantile g-computation model; WDI: weighted deprivation index.

^d^From the quantile g-computation model including the presence of foreigners in the area.

This effect seems to be slightly stronger among children aged 5-11 years, where the chances of being vaccinated decreased by 12% (OR 0.88, 95% CI 0.86-0.91) for each quintile increase of the ADI, while the decrease corresponded to 8% among children aged 12-14 years (OR 0.92, 95% CI 0.89-0.95; Table 2).

**Figure 2:**
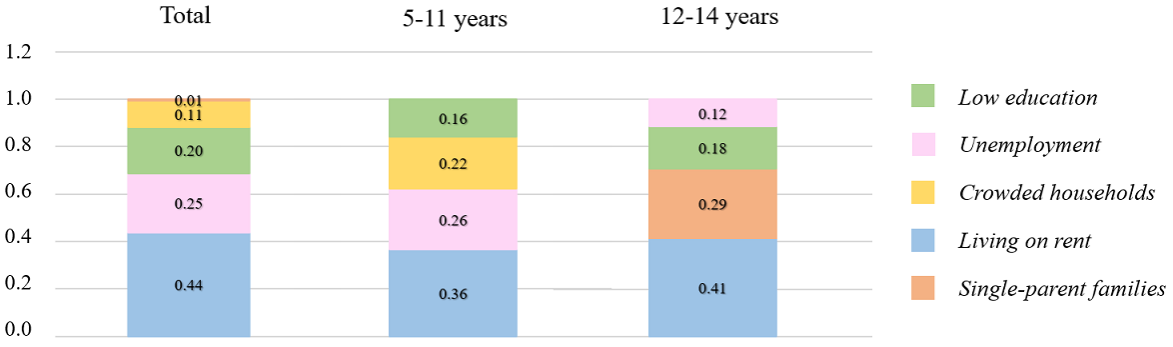
Quantile-g-computation model regression index negative weights for the probability of being vaccinated.

When assessing the effects of the WDI, a quintile increase was associated with a decrease in the probability of being vaccinated of 17% (overall OR 0.83, 95% CI 0.80-0.86; Table 2). The most influential condition on the probability of being vaccinated was living on rent (weight –0.44), which showed the highest weight in both 5-11–year-old children (weight –0.36) and 12-14–year-old children (weight –0.41; Figure 2). Being unemployed was also influential both overall and among the 5-11–year-old children (weights –0.25 and –0.26, respectively), while among 12-14–year-old children, single-parent families weighed the most (weight –0.29; Figure 2).

When including the prevalence of foreigners in the area among the area-based socioeconomic variables, no significant differences were observed in the WDI estimates (overall OR 0.82, 95% CI 0.79-0.85; Table 2). Nevertheless, on observing the weights of single indicators, a stronger association was found among younger children; the multiracial area was always identified as an influential condition, with the greatest weight, compared to the other variables both in the overall population and among children aged 12-14 years (weights –0.45 and –0.64, respectively; Table S3 in Multimedia Appendix 1). WDI analysis confirmed that living on rent remained the greatest determinant in the association of socioeconomic deprivation with COVID-19 vaccination in 5-11–year-old children (weight –0.24).

## Discussion

### Principal Findings and Comparison With Prior Work

The findings of this study have shown a significant reduction in the likelihood of receiving a COVID-19 vaccine among children living in areas characterized by greater socioeconomic deprivation. The dose-response gradient was strongest among children aged 5-11 years, for whom social and material deprivation reduced the chances of being vaccinated the most. The robustness of these findings is confirmed by results that were consistent across multiple analyses and definitions of the deprivation index.

Previous studies, mainly conducted in the United States, have shown how social inequalities affect COVID-19 vaccination rates in children [14-21]. To the best of our knowledge, this is one of the first studies to examine the association between a robust and largely used ADI and the registered vaccination status against COVID-19. Most other studies examining this association were surveys and mainly focused on parental willingness to vaccinate their children [14-16,18,20,21]. Only a few studies examined the COVID-19 vaccination status of children [17-19,22], but in most cases, data were reported by the parents [17-19].

When examining single indicators of lower socioeconomic status, items that were most negatively associated with the chances that a child received a COVID-19 vaccine were lower education [16,18-20], low income levels [14,18,20], high unemployment rates [16], living on rent [15], as well as living in a multiethnic area [14,18].

Comparable results to ours were reported by a study conducted in the United Kingdom based on over 27,000 students aged 9-18 years, which showed that the hesitancy toward vaccinating their children among both undecided parents (n=1688) and those against vaccination (n=4874) was higher among children in the most deprived quintile [15]. Regarding studies on reported vaccination status, in a large US survey of 59,424 children aged 12-17 years, Nguyen et al [19] found greater vaccination coverage among children whose parents had more than college education (adjusted prevalence ratio 1.15, 95% CI 1.05-1.25) [19]. Furthermore, a recent study elucidated variables associated with COVID-19 vaccination among Israeli adolescents (n=43,919) using data from an electronic database, showing that adolescents of low SEP had a very low vaccination rate (42.3%) compared to those having a high SEP (80.4%), which was highly significant upon univariate analysis but did not reach statistical significance using a multivariate approach [22].

Vaccination of children and adolescents largely depends on the vaccine hesitancy or antivaccination positions of the parents. Lower parental SEP has been associated with a lower parental propensity to vaccinate their children. In Europe, over the past decades, the link between SEP and vaccine coverage has been weakening, and a much more relevant role seems to be played by the lack of vaccine confidence or alternative health beliefs [34]. In the present mutating context, the pandemic may have determined hardly predictable effects. The increase in the vaccine coverage rate may have been primarily driven by the fear of infection and long-term consequences of COVID-19, while vaccine hesitancy may have increased as a result of the fear of adverse effects for newly registered vaccines, which may have been greater due to the novelty of mRNA vaccines [35-37]. Public perception of the pandemic and of COVID-19 vaccines was extremely heterogeneous. Mass vaccination campaigns and government incentives such as vaccine passports, which had not been implemented for decades in high-income countries, may have also affected vaccine hesitancy in different ways [38,39]. Nonetheless, it remains unclear how these opposing tendencies may have affected the association between SEP and COVID-19 vaccination.

When assessing the association between interquintile changes in the deprivation index and the likelihood of receiving a COVID-19 vaccine, the effects estimated from the WDI were always stronger. The ADI treats each dimension as if they were of equal importance by summing up their relative contribution with equal weight [27], rather than weighing indicators in accordance with their association with the outcome. This approach also allowed us to assess the role of the ethnic composition in each area, which was not included in the ADI. This variable was strongly associated with the likelihood of being vaccinated when considered individually as a socioeconomic determinant, but its inclusion in the WDI did not change the magnitude of the association of each quintile with the chances of being vaccinated, although this component weighted more than the others. This helps confirming the robustness of the national deprivation index (ie, ADI) to the inclusion of such a component.

We observed large differences in vaccination coverage by age, with much higher percentages among older children. A reasonable explanation could be found in the Italian COVID-19 regulations, issued differently in accordance with age: all adolescents aged ≥12 years had to exhibit a vaccine passport (a certificate provided to all people immunized against COVID-19) in order to participate, to most public, leisure, and sport activities, while there were no restrictions for younger children. This may also have contributed to a reduction in the role of SEP on the likelihood of vaccination specifically in adolescents, flattening the differences in this group. We can expect that this effect could be more evident in older adolescents (aged 15-19 years), who exceeded the maximum age required to be followed up by a FP. As age increases, the parental willingness to vaccinate their children may play a weaker role, as well as the SEP itself, given a greater autonomy of the adolescent.

Our study also showed how FPs were among the most relevant elements in determining the chances of receiving a COVID-19 vaccine. This suggests that promoting vaccination campaigns and raising awareness of the importance of pediatric vaccinations may significantly affect vaccination coverage among the children they assist.

### Strengths and Limitations

Our study has several strengths. First, the large cohort study was based on more than 25,000 children with real data on the vaccination status, regardless of the willingness or hesitancy to get a COVID-19 vaccine. Vaccinations were entirely managed by the National Health Service, and all vaccinations were promptly recorded in a centralized register, so data on vaccination coverage were complete and constantly updated. Data on previous SARS-CoV-2 infections that determined changes in the vaccination schedule based on government indications allowed us to perform more accurate analyses, thereby including only children who did not receive a COVID-19 vaccine for reasons likely not related to vaccine indications. Furthermore, we controlled by design the interpediatrician variability, which emerged as one of the main determinants of receiving a COVID-19 vaccination in our study population.

Another strength of this study was addressing the presence of intercorrelation between social determinants by using an ADI, both nonweighted and weighted, through a data-driven method for modeling the exposure to multiple socioeconomic determinants. This method provided a measure of the simultaneous effect of the included socioeconomic indicators after a quantile transformation, which makes the exposure more robust to extreme values and more comparable to other settings, as opposed to using value-based cutoffs [40]. This supervised approach empirically examines single contributions of each indicator to the association, allowing us to understand which condition most influences the likelihood of receiving a vaccine.

The main limitation of this work is the absence of comprehensive individual information on socioeconomic status, which is why a census block–level deprivation index was used. Still, we know that ADI represents a simplification that may not exactly correspond to the reality, but it acts as a good proxy of individual socioeconomic status or as a comparative measure of socioeconomic background [27].

A possible bias could derive from the exclusion of some FPs that did not adhere to the COVID-19 data registry linkage or that were missing up-to-date information about the children they assisted. However, we believe that this bias should be negligible, since the distribution of the ADI quintiles among the children included in the study did not differ from the regional ADI quintile distribution, suggesting that the study population is likely to be representative of the regional population.

Moreover, some residual confounders related to specific indications to not vaccinate children (ie, due to specific health conditions) cannot be entirely ruled out. However, the magnitude of the association between the likelihood of being vaccinated and the deprivation indexes we used makes an overturning of the results unlikely.

The relatively short study period also is unlikely to have affected our results, given the low vaccination rates in children from April 2022 onward.

### Conclusions

In a rapidly evolving pandemic scenario, achieving good vaccination coverage in adults is crucial, and high vaccination coverage could become a priority among children as well. It is of utmost importance for public health experts to identify population strata at risk of not achieving adequate vaccine coverage in order to remove barriers to accessing vaccines and to promoting targeted campaigns for these groups at increased risk. The relevance of vaccine coverage applies to COVID-19 vaccines, but also to other vaccines such as those against seasonal influenza.

## Appendix

Multimedia Appendix 1 Supplementary Figures and Tables.

Multimedia Appendix 2 Details on linkage with healthcare registries of the Veneto Region.

## Abbreviations

ADI: area deprivation index
FP: family pediatricians
OR: odds ratio
SEP: socioeconomic position
WDI: weighted deprivation index

## Data Availability

The data that support the findings of this study are available on request from the corresponding author (CC). The data are not publicly available due to restrictions (containing information that could compromise the privacy of research participants).
