# Supplementary material for: The Association Between Pediatric COVID-19 Vaccination and Socioeconomic Position: Nested Case-Control Study From the Pedianet Veneto Cohort
Source: JMIR Public Health Surveill. 2023 Feb 1;9:e44234. doi: 10.2196/44234 (PMC9897308; doi:10.2196/44234)
Supplement: Multimedia Appendix 1 [file publichealth_v9i1e44234_app1.pdf]

Figure S1. Flowchart of the study cohort (n=25,635).

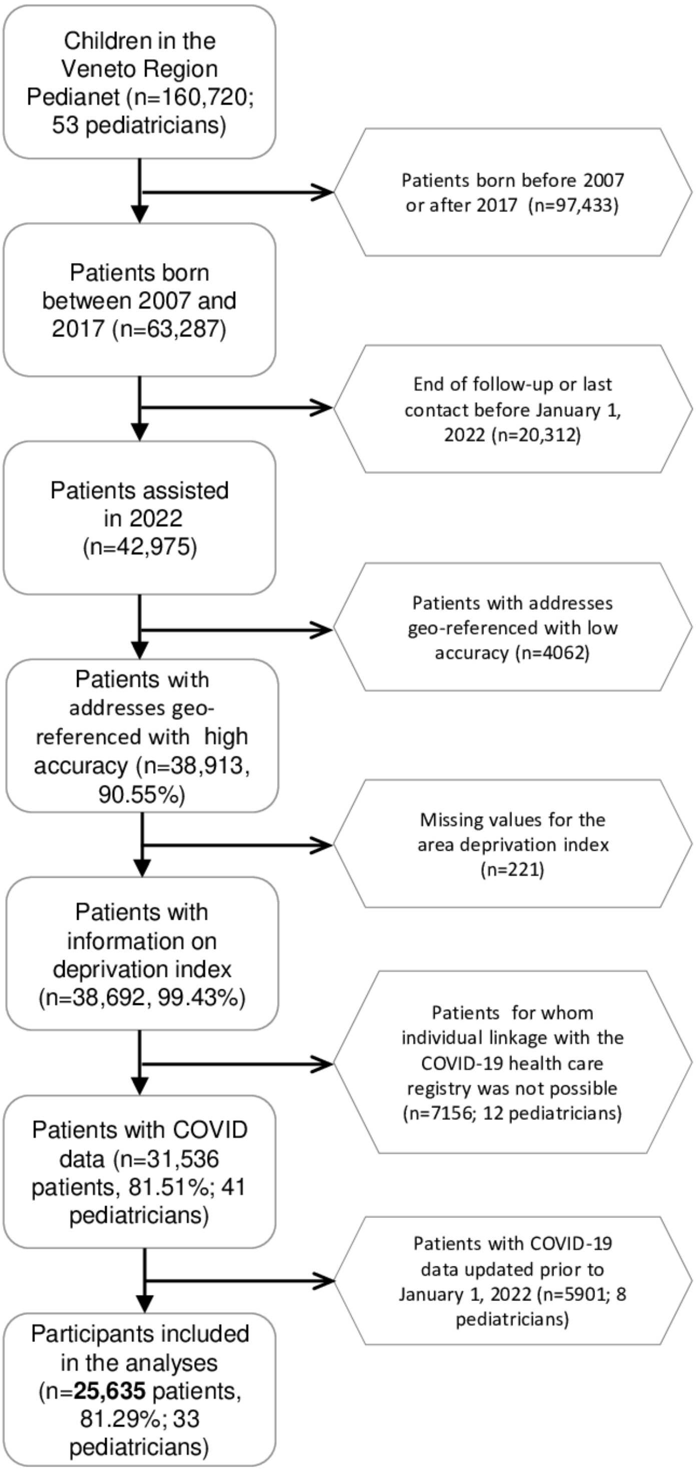

Table S1. Distribution of children characteristics, stratified by vaccination status at the end of follow-up (n=25,635).

Characteristics	All	Vaccinated (n=6,491)	Non Vaccinated (n=19,144)
Birth year (n,%)			
2007-2010	9505	3510 (36.9)	5995 (63.1)
2011-2013	8145	1660 (20.4)	6485 (79.6)
2014-2017	7985	1321 (16.5)	6664 (83.5)
Gender (n,%)			
Female	12358	3101 (25.1)	9257 (74.9)
Male	13277	3390 (25.5)	9887 (74.5)
Family pediatrician (n,%)			
2	770	348 (45.2)	422 (54.8)
16	838	151 (18.0)	687 (82.0)
17	817	297 (36.4)	520 (63.6)
30	872	312 (35.8)	560 (64.2)
35	903	439 (48.6)	464 (51.4)
39	922	245 (26.6)	677 (73.4)
55	1039	98 (9.4)	941 (90.6)
66	900	321 (35.7)	579 (64.3)
78	630	225 (35.7)	405 (64.3)
134	676	167 (24.7)	509 (75.3)
142	891	134 (15.0)	757 (85.0)
260	911	340 (37.3)	571 (62.7)
277	920	140 (15.2)	780 (84.8)
370	684	115 (16.8)	569 (83.2)
379	788	94 (11.9)	694 (88.1)
493	637	87 (13.7)	550 (86.3)
839	693	218 (31.5)	475 (68.5)
1790	642	139 (21.7)	503 (78.3)
2078	606	86 (14.2)	520 (85.8)
2167	770	272 (35.3)	498 (64.7)
2742	503	189 (37.6)	314 (62.4)
2808	832	100 (12)	732 (88)
2992	889	383 (43.1)	506 (56.9)
3118	672	167 (24.9)	505 (75.1)
4249	627	54 (8.6)	573 (91.4)
4630	829	155 (18.7)	674 (81.3)
4738	797	121 (15.2)	676 (84.8)
4916	738	253 (34.3)	485 (65.7)
4942	567	225 (39.7)	342 (60.3)
5089	1144	116 (10.1)	1028 (89.9)
5988	570	113 (19.8)	457 (80.2)
6005	653	211 (32.3)	442 (67.7)
7528	905	176 (19.4)	729 (80.6)

Table S2. Area Deprivation Index quintiles distribution, stratified by vaccination status at the end of follow-up (n=25,635).

**Area Deprivation Index quintile (n,%)**	**All**	**Vaccinated (n=6,491)**	**Non Vaccinated (n=19,144)**
1	5320 (20.8)	1508 (23.2)	3812 (19.9)
2	5077 (19.8)	1334 (20.6)	3743 (19.6)
3	4830 (18.8)	1237 (19.1)	3593 (18.8)
4	5443 (21.2)	1285 (19.8)	4158 (21.7)
5	4965 (19.4)	1127 (17.4)	3838 (20.0)

Table S3. Quantile G-computation (Q-Gcomp) model regression index weights for the probability of being vaccinated, including the condition of being foreigner in the WDI.

	**Weigths[a]**							
	**Direction**	**Effect**	*low education*	*unemployment*	*crowded households*	*living on rent*	*single-parent families*	*foreigners*
**Total**	pos	1.00					1	
	neg	0.82	0.15	**0.18**	0.06	0.15	0.01	**0.45**
**5-11yo**	pos	1.04					1.00	
	neg	0.78	0.14	**0.23**	0.20	**0.24**		**0.21**
**12-14yo**	pos	1.03			0.76	0.24		
	neg	0.80	0.10	0.02			0.24	**0.64**

[a]In bold weights that exceed the case of uniform negative weights (>1/p, where p represents the number of socioeconomic variables with negative weights in each group).
