# Supplementary material for: The Association Between Pediatric COVID-19 Vaccination and Socioeconomic Position: Nested Case-Control Study From the Pedianet Veneto Cohort
Source: JMIR Public Health Surveill. 2023 Feb 1;9:e44234. doi: 10.2196/44234 (PMC9897308; doi:10.2196/44234)
Supplement: Multimedia Appendix 2 [file publichealth_v9i1e44234_app2.docx]

§ The linkage with the healthcare registries of the Veneto Region was made through the Junior Bit® software, the system used by FPs to collect the clinical information on the pediatric primary care database Pedianet. To avoid the misclassification of the exposure of interest, we included in the cohort only those children of FPs who downloaded the information on vaccination and swab at least March 2022. For this reason, a huge number of pediatricians were excluded from the cohort.
